# Effect of copper mill waste material on benthic invertebrates and zooplankton diversity and abundance

**DOI:** 10.1371/journal.pone.0318980

**Published:** 2025-03-03

**Authors:** James H. Larson, Michael R. Lowe, Sean W. Bailey, Amanda H. Bell, Danielle M. Cleveland

**Affiliations:** 1 U.S. Geological Survey, Upper Midwest Environmental Sciences Center, La Crosse, Wisconsin, United States of America; 2 U.S. Geological Survey, Great Lakes Science Center, Hammond Bay Biological Station, Millersburg, Michigan, United States of America; 3 U.S. Geological Survey, Upper Midwest Water Science Center, Madison, Wisconsin, United States of America; 4 U.S. Geological Survey, Columbia Environmental Research Center, Columbia, Missouri, United States of America; Tsinghua University, CHINA

## Abstract

Copper (Cu) stamp mill mining in North America from the early 1900s produced a pulverized ore by-product now known as stamp sands (SS). In a mining operation near the city of Gay (Michigan, USA), SS were originally deposited near a Lake Superior beach, but erosion and wave action have moved many SS into beaches and reefs that are critical spawning and nursery areas for native fish (e.g., Lake Whitefish). Larval and juvenile native fish consume zooplankton and benthic invertebrates during their development, and many of these invertebrate taxa may be sensitive to metal contamination from the SS. Here, we sampled the invertebrate community from beaches with high SS, moderate SS and low SS, as well as a control beach 58 km from the source of the SS. The high SS site was characterized by fewer benthic taxa, and less density of several taxa than the low SS site, especially benthic copepods. All beaches had comparable zooplankton diversity, but the abundance was ~ 2 orders of magnitude lower at the high SS site. Cu and several other metals were elevated at beaches with more SS. We found support for associations between benthic density and diversity with depth (positive effect) and Cu concentration (negative effect). Cu concentration was a better predictor of declines in benthic invertebrate abundance and diversity than SS although sensitivity to Cu varied among taxa. We also observed that the relationship between Cu concentration and SS was non-linear, and highly variable. For example, 149 mg Cu/kg dry weight sediment is a consensus threshold used in the literature to identify Cu toxicity, but the prediction interval for estimating that concentration of Cu from measurements of SS is 26-851 mg Cu/kg dry weight. A better predictive model of this relationship would be beneficial to develop an understanding of what level of SS reduction would prevent Cu impacts on invertebrates.

## Introduction

Historical or legacy mining activities may leave long environmental legacies. Historical copper mining in the watersheds surrounding Lake Superior was done by bringing ore-bearing rock to stamp mills, that crushed the rock into a consistent size for copper removal. These ‘stamp sands’ (SS) were discharged to nearby lakes, rivers and streams [1–3]. One location where SS were deposited was on the eastern shoreline of the Keweenaw Peninsula, Lake Superior near Gay, Michigan. Between 1900 and 1932, approximately 22.7 million metric tons (Mt) of SS was discharged to the beach near the mill [4]. Of the original 22.7 Mt, an estimated 11 Mt of SS remains on the shoreline, and the remainder (minus approximately 1.4 Mt removed by the local road commissions for use as traction sand) have been moved by wave and wind southwest along the shoreline and outward into Grand Traverse Bay, threatening Buffalo Reef [3,5,6].

Buffalo Reef is a 2,200-acre cobble and bedrock formation that is situated 5 km south of the original pile of SS in 3–30 m of water and less than 100 m from the SS-covered beach. The reef has long been recognized as an important spawning habitat for Lake Trout (*Salvelinus namaycush*) and Lake Whitefish (*Coregonus clupeaformis*) [7,8]. Early work on SS determined that not only do SS contain copper (Cu) in concentrations that are toxic to cold-water fishes [9], but also that > 25% of Buffalo Reef’s surface was covered with SS as of 2009 [5]. By 2015, the extent of the reef covered by SS had increased to 35% [5], and hydrodynamic models predicted that 60% of the reef would be inundated by SS by 2026 if no management action was taken [10].

Routine monitoring and assessment efforts have demonstrated declining numbers of spawning Lake Trout and Lake Whitefish in recent years [11]. Young-of-year (YOY) Lake Whitefish in the beaches immediately adjacent to Buffalo Reef are absent and there have been sharp declines in YOY Lake Whitefish abundance in the beaches in the southern portion of Grand Traverse Bay since 2008 [12]. The mechanisms driving these observed changes are poorly understood. Stamp sands can exert both chemical and physical changes to the ecosystem. The drifting SS have physically altered the surface of the reef and smothered its nearshore area [3] where Lake Whitefish once spawned [8]. Even in areas where the surface of the reef is not inundated, SS have likely settled into the interstitial spaces that are critical to the survival of the early life-stages of Lake Whitefish [13]. Moreover, *in situ* conditions within the reef are difficult to assess, due to the water depths and temporal variations in SS presence (e.g., drifting, leaching, weathering).

Many common prey items for larval and juvenile fishes [14,15] can be sensitive to metals, resulting in effects such as reduced populations or extirpation. In a pair of studies, Kerfoot et al. [3,5] summarized several years of field data and found that both taxonomic diversity and abundance of benthic macroinvertebrates are negatively affected by SS. Reduced survival and growth of Amphipoda and Cladocera was also observed in a laboratory study in response to exposure to SS elutriates [16]. Zooplankton and benthic invertebrates can also take up metals via ingestion of contaminated algae, sediment, periphyton, and other detritus, which creates another exposure pathway for fish.

Here we hypothesized that invertebrate communities in the beach environments that support larval and juvenile fish are negatively impacted by the presence of SS. We hypothesized these impacts are primarily related to metal toxicity, and that zooplankton will be more sensitive than benthic invertebrates to these metal effects.

## Methods

### Study Area

**Fig 1:**
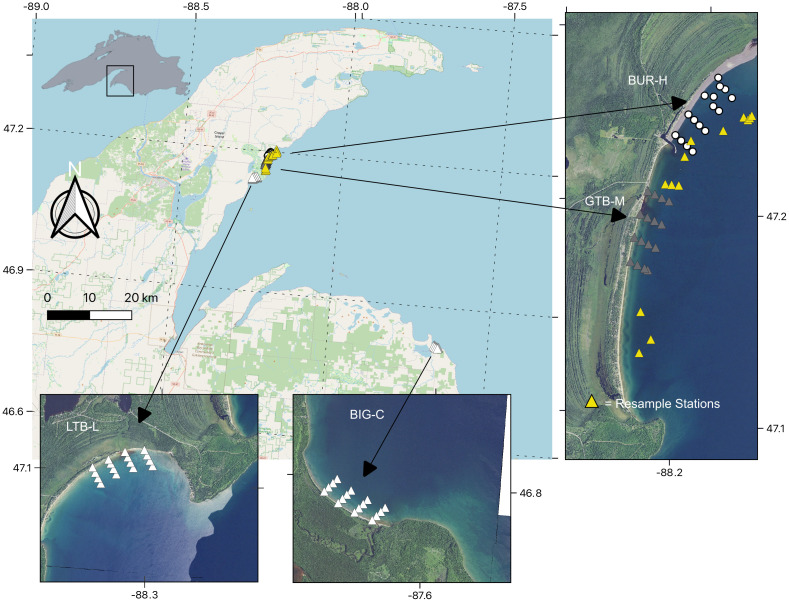
Map showing beaches and approximate sites sampled for benthic invertebrates in Lake Superior near the Keweenaw Peninsula, Michigan, 2021. Samples were collected to assess the association between stamp sands (SS, a byproduct of copper mining) and invertebrate communities. At each site, a petite Ponar was used to collect sediment, which was analyzed for benthic invertebrates, metal concentrations and SS percentage. The beach most highly contaminated with SS is near Buffalo Reef (BUR-H; white circles). Another beach in Grand Traverse Bay that has been protected by a pier south of BUR-H is known to have some SS contamination (GTB-M; grey triangles). Further south and around a bend in the shoreline is another bay, where we did not expect to observe SS, but we did observe low concentrations of SS ((Little Traverse Bay; LTB-L; white triangles). A control beach with was located ~ 58 km away (Big Bay; BIG-C; white triangles). In addition, we sampled some sites previously sampled by Kerfoot et al. ( [5]; Resample stations; yellow triangles). Base map data from OpenStreetMap (openstreetmap.org/copyright), and imagery is from the U.S. Geological Survey (https://www.usgs.gov/tools/national-map-viewer).

Our study area consisted of four beaches spread across Keweenaw Bay, Lake Superior. Each beach had varying SS presence, based on previous mapping of the SS distribution by Kerfoot et al. [3] (Fig 1). Beaches are the focus of this study because these are important foraging areas for juvenile fish spawned on the nearby reefs. Two beaches were located in Grand Traverse Bay (Lake Superior), one adjacent to Buffalo Reef with high SS (BUR-H) and one south of the harbor breakwall at the Traverse River with moderate SS (Grand Traverse Bay beach; GTB-M) (Fig 1A). The breakwall was installed in 1950 to provide a safe harbor for boats; however, it also reduced the movement of the SS and, until recently, prevented SS intrusion at GTB-M. The drifting SS eventually overtopped the wall and recently low amounts (<15% in the nearshore environment) of SS have been documented in some areas of GTB-M [3]. Conceptually, BUR-H represents a site that has been substantially inundated by SS, whereas GTB-M has experienced comparatively fewer SS to date. In addition to the beach sampling areas in Grand Traverse Bay, we resampled 16 sites across the bay (Fig 1) that had previously been reported (RES) in Kerfoot et al. [5].

Two other sites were sampled with low or no SS influence. Continuing southwest along the Keweenaw Peninsula is a beach in Little Traverse Bay that we expected to have no SS influence (LTB-L). Given the distance of LTB-L from Gay, Michigan, and the orientation of the shoreline (i.e., LTB-L is shielded from SS migration by Traverse Point to the north/east; Fig 1B), this site is likely to have very low SS relative to BUR-H, although given the direction of SS movement we knew there was a possibility of SS reaching LTB-L [3]. Prior to this study, we assumed that LTB-L would be a control site, however since we observed small amount of SS in LTB-L, we will refer to it from here-on as a low-impact site (hence the L in the beach abbreviation). Instead, our only control site was the beach at Big Bay (BIG-C), which is approximately 58 km from the original SS pile, on the opposite side of the Keweenaw Bay near Big Bay, Michigan. The BIG-C site is unlikely to receive SS from the pile at Gay, Michigan, due to the overall distance separating the two locations. A nickel-copper mine is currently operating in the BIG-C watershed, but it has a relatively small footprint (surface operations: 53 ha) and does not produce SS. There are no other records of SS on the Lake Superior shoreline along the southern edge of Keweenaw Bay or in the vicinity of BIG-C. This site also has a different orientation than the other sites, ‘facing’ north/northeast (N/NE) (out of Keweenaw Bay), whereas the other beaches face south/southeast (S/SE) (the opposite side of Keweenaw Bay). As a result, the beach at BIG-C is more exposed to wind and wave action from the main fetch of Lake Superior, which may affect the invertebrate community.

### Sediment and macroinvertebrate sample collection

As no vertebrates or protected taxa were targeted in this study, no permits were required for the sampling conducted in this study. The sites are on navigable waters and therefore site access is not restricted.

At each beach, we collected sediments and benthic macroinvertebrates at 16 stations that were stratified across depth. A nearshore, shallow-water sample site was selected randomly from along the beach and 3 deeper sites were selected along a line roughly perpendicular to the shoreline that ended at either roughly 9 m or at the point where the bottom flattened out or began to rise back up. Exact locations for the different depths were not targeted *a priori* and coordinates are accurate only to within about 30 m due to wind and wave action moving the boat around the anchor (Fig 1B, C, and D). Sediment and benthic invertebrates were collected between August 17 and August 19, 2021, for BUR-H, GTB-M, LTB-L and RES samples, and on August 23, 2021, for BIG-C. A single sample from each site was collected with a petite Ponar grab sampler (Wildco [17];) and separated into sediment and macroinvertebrate fractions in the field. The Ponar sampler was thoroughly cleaned between each sampling site. Each sediment sample was mixed well on the boat, and a subsample of sediment (approximately 65 g) was collected and split for grain size analysis, including estimation of percent SS, and analyses of total recoverable metals. Subsamples were frozen until further processing. The remaining portions of the grab sediments were then sieved (500-µm mesh) to remove small debris and capture benthic invertebrates [18]. Material not passing through the sieve was decanted into plastic bottles filled with 10% neutral buffered formalin (NBF) and a Rose Bengal dye for macroinvertebrate identification and quantification. Water temperature (°C), dissolved oxygen (mg/L), and pH were recorded at each site at which sediment and benthic invertebrates were collected (just above the water-sediment interface).

### Sediment processing

Sediment sub-samples for SS determinations were taken to the U.S. Geological Survey’s (USGS) Hammond Bay Biological Station, thawed in the laboratory, oven-dried to a constant temperature at 65 °C, and then passed through a series of sieves (4-mm, 2-mm, 500-µm, 250-µm, 125-µm, and 63-µm) for grain size analysis. To estimate the amount of SS in each sediment, three replicate samples were collected by pressing double-sided tape into the surface of the dried sample (after thorough mixing). This tape was then affixed to a glass microscope slide with an etched, 9 x 9, labelled grid (each grid cell was 1.0 mm x 1.0 mm); slides were imaged at 20x magnification using a 12-megapixel CMOS camera (AmScope) fixed to a compound microscope (Zeiss Primo Star). Four cells from each grid were randomly selected and the number of stramp sand and natural quartz sand grains were enumerated in each of the cells by two different individuals.

Sub-samples of frozen sediments for analyses of total recoverable metals were sent to the USGS Columbia Environmental Research Center in Columbia, Missouri. Arsenic (As), cadmium (Cd), chromium (Cr), cobalt (Co), copper (Cu), lead (Pb), iron (Fe), manganese (Mn), nickel (Ni), selenium (Se), thallium (Tl), and zinc (Zn) in the sediments were quantified using inductively coupled plasma-mass spectrometry (PerkinElmer Nexion 2000; similar to U.S. Environmental Protection Agency [US EPA] 6020B [19]) following microwave-assisted digestion in high purity nitric acid and 30% hydrogen peroxide (similar to US EPA method 3050B [20]). This digestion method accesses elemental concentrations that could become biologically or environmentally available; it is not a total digestion method. All sediment samples were lyophilized prior to digestion, and elemental concentrations were determined in units of milligram per kilogram on a dry weight basis (mg/kg DW). Quality control (QC) measures included the use of at least four NIST-traceable calibration standards, second-source initial and continuing calibration standards and blanks, and laboratory control standards, as well as method digestion blanks, digestion triplicates and spikes, and the use of certified reference materials having a soil or sediment matrix; all QC results were within acceptable limits for the method. The limits of quantification (LOQs) were the following (mg/kg dw): As, 0.04–0.1; Cd, 0.02–0.04; Co, 0.02–0.03; Cr, 0.04; Cu, 0.1–0.2; Fe, 1–2; Mn, 0.2–0.4; Ni, 0.1–0.2; Pb, 0.02; Se, 0.1; Tl, 0.02–0.03; and Zn, 0.3–0.4. Concentrations of Cr, Mn, Fe, Co, Ni, Cu, Zn, As, and Pb in all 79 sediments were above their respective LOQs. There were 31 sediments having Se concentrations < LOQ (39%) and 38 Cd concentrations < LOQ (48%); all 79 sediments had Tl concentrations < LOQ (100%).

### Macroinvertebrate processing

Benthic macroinvertebrate samples were processed at the USGS Upper Midwest Environmental Sciences Center in La Crosse, Wisconsin. The NBF/Rose Bengal dye solution was decanted and replaced with 95% ethanol to facilitate processing and identification. Using a zoom stereo microscope (7–70x magnification; Olympus SZH10), invertebrates were sorted and identified to at least the level of taxonomic classification used in Kerfoot et al. [5]. In most cases, this was to the Family level, but some taxa were identified down to genus or species [21–23].

### Zooplankton sampling and processing

Zooplankton were sampled on three dates at each beach (between June 21 and July 25, 2021) by towing a nylon plankton net (153-µm mesh; 50-cm diameter x 150-cm long opening [24]) parallel to the shoreline along shallow (0.76–2.23 m) and deep (3.14–5.30 m) water zones. Two replicate, 5-minute tows were performed in each depth zone, and both shallow and deep tows were added together into a composite sample. Tows were 30 m behind the vessel; distance traveled (m), tow speed (cm/s), and water volume filtered (cm^3^) were recorded with a mechanical flowmeter mounted in the center of the net ring. Upon collection, the net was washed down with filtered lake water (from the outside of the net); captured zooplankton were stored in 95% ethanol and shipped to a contract laboratory for identification and enumeration (EcoAnalysts, Inc., Moscow, Idaho).

EcoAnalysts processed the zooplankton samples according to US EPA SOP LG403 Standard Operating Procedure for Zooplankton Analysis [25]. Briefly, a sub-sample of 200–400 microcrustaceans (except nauplii) was identified to the lowest practical level and enumerated. If aquatic organisms of interest were found, 3–5 organisms of each taxon were carefully removed from the sample and isolated for further inspection and reference. Samples were identified using the technical literature and reflects the accepted nomenclature.

### Statistical analysis

All statistical analyses, including determinations of standard descriptive statistics and simple correlations (e.g., Pearson’s *r*), were performed using R [26]. We used the non-parametric Peto-Peto comparison, as implemented in the R package NADA2 [27,28], to compare metal concentrations in sediments among beaches; this non-parametric method handles both non-normal data and censored data (e.g., concentration results < LOQ). The software also reports the median values for the groups, using Kaplan-Meier methods, which are appropriate for occasions where non-detects occur or distributions are not well known [29]. For statistical comparisons of metal concentrations among sites, we focused on the 10 metals that were both quantified in most samples and were enriched at BUR-H (thought to be due to a greater relative presence of SS) relative to the other sites: Cd, Co, Cr, Cu, Fe, Mn, Ni, Pb, Se, and Zn. As previously mentioned, Tl was < LOQ at all sites; As concentrations were relatively similar among all sites (n = 79; mean ±  standard error [SE], 1.68 ±  0.06 mg/kg dw).

We also characterized overall sediment toxicity due to metals by comparing our total recoverable results to consensus-based probable effects concentrations (PECs [30]). A PEC represents a concentration of a contaminant in a sediment (generally with particle size < 2-mm) above which adverse effects are probable. Metal PECs are based on total concentrations [31]; in this way, concentrations measured following our digestion method, which generally accesses only the fraction of metal(s) that could become environmentally or biologically available, may ultimately underestimate toxicity relative to the PECs. However, total recoverable concentrations have been used previously to screen for metal hazards (e.g., [32]). Individual PEC values (mg/kg dw) are the following: As, 33; Cr, 111; Cu, 149; Pb, 128; Ni, 48.6; and Zn, 459. Although there is a PEC for Cd (4.98 mg/kg dw), we did not include it in our assessments because Cd concentrations were generally near or < LOQ in all sediment samples; all samples had Cd ≤ 0.14 mg/kg dw, so Cd contributions to overall toxicity were likely small. There is currently no PEC for Co; however, we substituted a Canadian clean-up target (80 mg/kg dw) for the purposes of our comparisons [33]. No PECs are available for Mn, Fe, Se, or Tl, so these metals were not included in our sediment toxicity evaluations.

We also considered PEC quotients (PEQ) to assess the potential for additive effects of multiple metal stressors. A PEQ is the ratio of the measured concentration of metal to the PEC; a PEQ quantifies the degree to which a metal concentration in a sediment sample exceeds the PEC threshold. For example, Cu has a PEC of 149 mg Cu/kg dw, so a sediment sample with 745 mg Cu/kg DW has a PEQ of 5 (745 divided by the PEC). This is somewhat analogous to the US EPA’s Hazard Quotient and Hazard Index metrics for assessments of potential risks to human health resulting from exposure to chemical mixtures [34]. In multi-stressor situations, such as might be experienced by benthic communities at BUR due to the presence of multiple metals in the SS, overall metal hazards might usefully be estimated as ΣPEQ. In other words, a PEQ is calculated for each individual stressor to normalize the concentration to the relative toxicity of that metal; the PEQs are then summed (ΣPEQ) to generate a toxicity metric that assumes the hazards from individual metals are fully additive [32,35,36]. A ΣPEQ ≥ 1 is generally considered a conservative screening indicator of sediment toxicity to benthic organisms [36]. However, this approach does not consider potential synergistic and antagonistic effects wherein co-stressors interact to produce more-than-additive or less-than-additive toxicity [36,37], nor does it consider any physical effects that SS might have on biota (such as SS smothering interstitial spaces).

We used non-metric multidimensional scaling (NMDS, with two dimensions) to visualize community composition differences (using the vegan package in R [38]). NMDS is used to create a ‘map’ of differences among sites using some measure of distance or dissimilarity, which facilitates visual comparisons [38]. We used the default distance matrix in the vegan package (the Bray-Curtis dissimilarity index) on abundance data with the autotransformation option selected (in this case, Wisconsin double standardization after a square root transformation). After plotting the sites in a two-dimensional space, we plotted the 95% confidence intervals and the standard deviation around the centroid for each beach. The NMDS for benthic macroinvertebrates included Gastropoda, Sphaeriidae, Hydrachnidae, Trichoptera, Nematoda, Oligochaeta, Amphipoda, Chironomidae, Copepoda, and Cladocera. The NMDS for zooplankton included Acari, Bosminidae, Calanoida, Centropagidae, Cercopagididae, Chironomidae, Chydoridae, Copepoda, Cyclopidae, Cyclopoida, Daphniidae, Diaptomidae, Ephemeroptera, Harpacticoida, Holopediidae, Ilyocryptidae, Ostracoda, Polyphemidae, Sididae, and Temoridae. Each individual was only included in the lowest taxonomic classification in which it could be identified (e.g., a Diaptomidae individual is only included in the Diaptomidae group, not the Copepoda group).

We used a generalized linear model (in base R) with beach as a categorical predictor to assess differences in benthic community composition among beach areas. This is conceptually identical to a standard analysis of variance (ANOVA) method in that it identifies whether a categorical variable is associated with changes in a continuous response variable. The response variables we examined here included the density of benthic invertebrates, zooplankton and all individual taxa. Some taxa could not be compared using this approach due to their very rare occurrence (Trichoptera, Gastropoda, Hydrachnidae, Nematoda among taxa identified in benthic samples; Chironomidae and Ephemeroptera among zooplankton samples). When using categorical predictors, one category is used as the default, and the effect of switching from that category to another category is estimated. In our models, we used BUR-H as our default category so that we could compare other beach areas to it, since we know from other studies that this area is heavily covered by SS [5]. Standardized slopes (β) and the coefficient of determination (R^2^) values were calculated as standardized estimates of effect size [39]. Because RES samples were collected with a different sampling approach, they are not included in any of the among-beach comparisons.

We used multi-level models (R package lm4e) that included the beach as a random effect on the intercept to assess the direct relationship between SS and benthic community composition (invertebrate density and number of taxa). Sites resampled from Kerfoot et al. ( [5]; RES) were treated as a separate group of sites because they represented a distinct sampling strategy. Kerfoot et al. [3,5] had previously found that the model that best fit the data included both SS and SS squared (SS^2^), so we also included a model with both SS and SS^2^. The following parameters were used to represent SS: SS, SS plus SS^2^, total recoverable Cu concentrations, and ΣPEQ; each of these SS parameters was included in a model that predicted either benthic invertebrate density (using a natural log distribution) or benthic invertebrate taxa count (using a Poisson distribution). Additional models included water depth, or a combination of water depth with an SS parameter. We also included a null model that included only the random effect of beach. A complete list of models (n =  12) considered is included in the results. For each model, the Akaike’s Information Criterion (corrected for small sample size; AIC_C_) value was calculated, and models were ranked according to the difference (Δ) in AIC_C_ values (lower ΔAIC_C_ values indicate a better-fit model [40]). Typically, ΔAIC_C_ is estimated relative to the best-fit model (i.e., the model with the lowest AIC_C_ value); models with a ΔAIC_C_ < 2 are considered to have a fit equivalent to the best model, while models with ΔAIC_C_ > 10 are usually considered to have much worse fit than the best model, although these are guidelines rather than objective criteria [40,41]. If two models have ΔAIC_C_ < 2, but one model has more parameters than the other, the model with fewer parameters is generally considered to have stronger support [42]. For the most strongly supported model, we estimated standardized slopes (β), and for all models we estimated marginal and conditional R^2^ values as standardized estimates of effect size [39,43,44]. Marginal R^2^ values indicate variation explained by the fixed effects (i.e., in this case variables such as water depth and SS), while conditional R^2^ values indicate variation explained by the marginal effects conditional on the random effects (i.e., the beach).

We also performed a community multilevel model comparison using the approach of Jackson et al. [45] The community analysis included all of the benthic taxa. In this approach, the abundance of individuals observed is treated as a function of the fixed effects (predictor variables) and taxa identity is treated as a random effect. Beach was also included as a random effect as in the earlier model to account for beach-specific differences in suitability. Rather than model presence/absence, as in Jackson et al. [45], we modeled the number of individuals in a petite Ponar sample. We attempted both a Poisson distribution and a negative binomial distribution for this modeling, and found that negative binomial distributions fit the data much better (based on AIC_C_ comparisons). As with the multi-level models described in the previous paragraph, we used AIC_C_ to rank models that used SS, SS plus SS^2^, total recoverable Cu concentrations, and ΣPEQ in combination with or without water depth to predict the number of individuals present in a petite Ponar sample.

## Results

### Stamp sand and metals concentrations across the beach areas

**Fig 2:**
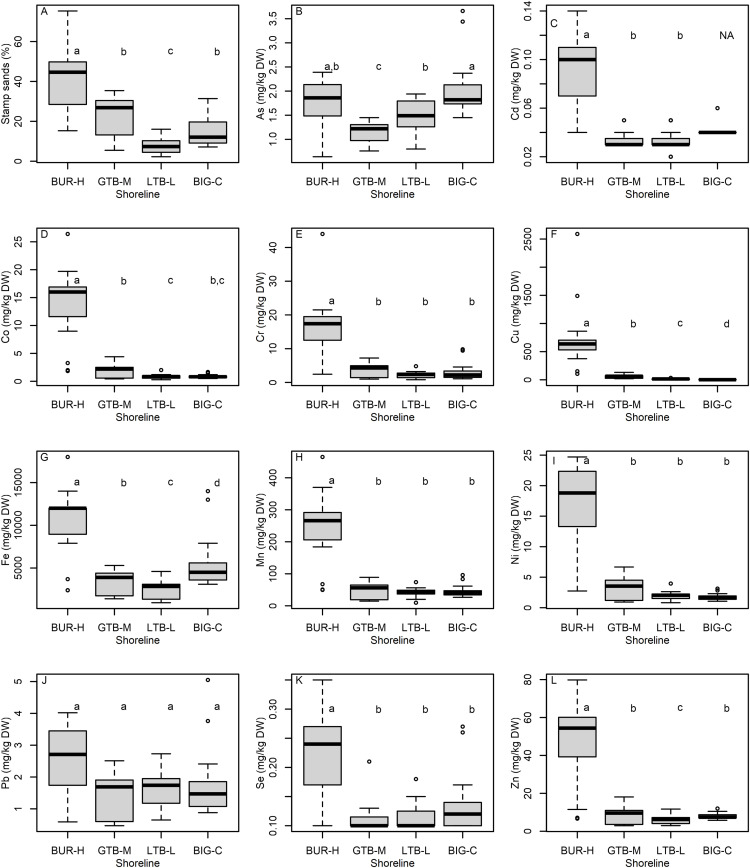
Graphical comparisons of (A) stamp sand content (percent) and total recoverable concentrations (mg/kg dry weight, DW) of (B) As, arsenic; (C) Cd, cadmium; (D) Co, cobalt; (E) Cr, chromium; (F) Cu, copper; (G) Fe, iron; (H) Mn, manganese; (I) Ni, nickel; (J) Pb, lead; (K) Se, selenium; and (L) Zn, zinc; in the sediments collected by petite Ponar from beach sites at Buffalo Reef (BUR-H), Grand Traverse Bay south of Traverse River (GTB-M), Little Traverse Bay (LTB-L) and Big Bay (BIG-C), Michigan in 2021. Letters in individual panels refer to beaches that were not clearly different using the non-parametric Peto-Peto test. In all panels, boxes encompass the first and third quartiles. The lines (whiskers) show the largest or smallest observation that falls within 1.5 times the box size. Observations that fall outside the lines are shown individually.

Beaches had similar temperature, dissolved oxygen and pH characteristics, but varied substantially in terms of water depth and the range of depths sampled (Table 1). Visual estimates of SS percentage indicated SS were more prevalent at BUR-H, lower at GTB-M, and lowest at LTB-L, as evidenced by both increased percentages of SS (Fig. 2A) and greater metals concentrations in the sediment samples at BUR-H relative to the other sites (Fig 2, Table S1). We noted dark particles present in the BIG-C beach samples that were visually similar to SS. However, these particles are unlikely to be SS from the pile at Gay, Michigan, based on the relatively low concentrations of metals typically characteristic of the Gay SS (e.g., Cd, Co, Cr, Cu, Mn, Ni, Zn) relative to BUR-H (Fig 2); thus, we believe the black particles in BIG-C sediments were from another source (e.g., manganese sands can be confused for SS [3],). Although manganese sands are only a small fraction of the sands at Grand Traverse Bay, they may be more prevalent on other beaches [3].

**Table 1:** Aggregated physical and water quality characteristics of sampling areas where benthic invertebrates (all sites) and zooplankton (beach sites) were collected in 2021 to assess the impact of stamp sands (in Lake Superior near the Keweenaw Peninsula, Michigan). Means are reported with standard deviation.

Sample group^*1*^	Mean Water Depth (m)	Minimum Water Depth (m)^2^	Maximum Water Depth (m)^2^	Water Temperature (°C)	Dissolved Oxygen (mg/L)	pH
Buffalo Reef Beach (BUR-H)	4.59 (1.8)	1.22	7.65	19.4 (0.7)	9.36 (0.9)	8.22 (0.1)
Grand Traverse Bay Beach (GTB-M)	4.11 (2.7)	0.73	8.20	19.0 (0.4)	9.48 (0.5)	8.21 (0.1)
Little Traverse Bay Beach (LTB-L)	2.55 (1.4)	0.61	4.66	18.9 (0.3)	9.28 (0.4)	8.19 (0.1)
Big Bay Beach (BIG-C)	3.65 (2.7)	0.61	7.65	19.8 (2.7)	8.74 (0.3)	8.23 (0.1)
Resampled sites (RES, in Grand Traverse Bay)	7.64 (1.3)	5.21	9.48	18.8 (0.8)	9.86 (0.7)	8.28 (0.1)

^1^Resampled sites include 16 sites that were previously sampled in Kerfoot et al. [5]. All other beaches were sampled along four transects, each consisting of four individual locations, across a gradient from shallow to deep water.

^2^Water depths at the shallowest and deepest points along the transects are reflected as the minimum and maximum depths, respectively.

**Fig 3:**
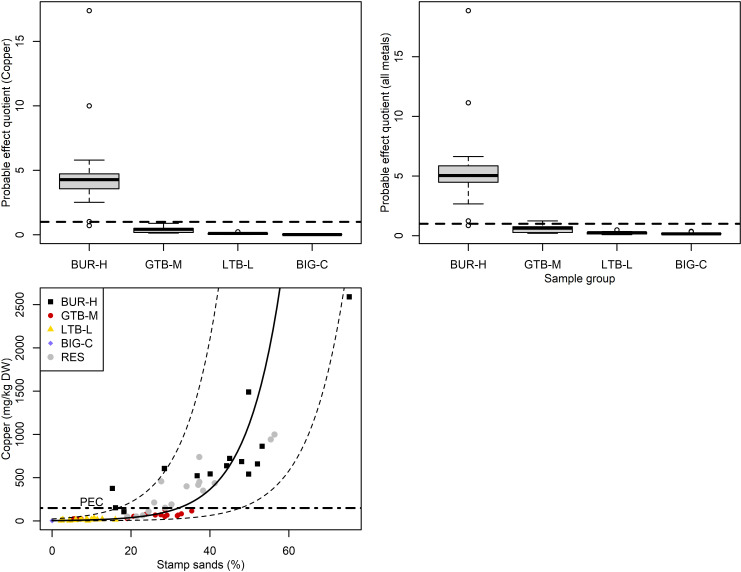
Probable effects quotients (PEQs) for total recoverable (A) copper and (B) sum of 7 metals (as ΣPEQs) in the sediments collected bypetite Ponar from study sites in Lake Superior near the Keweenaw Peninsula, Michigan, 2021. The ΣPEQ calculations in panel B include the PEQ for copper, arsenic, chromium, lead, nickel, zinc, and cobalt; the horizontal dotted lines in panels A and B are PEQ = 1, above which increased rates of adverse biological effects are expected. In panels A and B, boxes encompass the first and third quartiles. The thick black line is the median. The lines (whiskers) show the largest or smallest observation that falls within 1.5 times the box size. Observations that fall outside the lines are shown individually. (C) The bivariate relationship between stamp sands (as % of sediment) and copper concentration; the horizontal dotted line shows the probable effects concentration (PEC) for copper (149 mg/kg dw, equivalent to a PEQ of 1), above which biological effects are considered likely. The solid line (panel C) is the Cu concentration predicted from a model relating stamp sands to the log of the copper concentration (dashed lines indicate 95% confidence interval on the prediction). BUR-H – beach near Buffalo Reef that has been heavily impacted by stamp sands (SS); GTB-M – beach in Grand Traverse Bay south of the Traverse River with some SS contamination; LTB-L – beach in Little Traverse Bay with very little SS; BIG-C- beach in Big Bay (a control site with no SS); RES – samples from sites resampled from Kerfoot et al. [5].

Sediments from the BUR-H beach were enriched in Cd, Co, Cr, Cu, Fe, Mn, Ni, Se, and Zn (Fig 2, Table S1). Individual metal PECs were only exceeded for Cu (i.e., PEQ ≥ 1), and then only in samples from BUR-H or RES (Fig 3), which had broad overlap with BUR-H. When PEQs were summed across 7 metals (ΣPEQ), sediment samples from 14 of 15 BUR-H sites, 13 of 16 RES sites, and 2 of 16 sites at GTB-M had a ΣPEQ ≥  1 (Fig 3). No BIG-C or LTB-L sites had PEQs ≥ 1 or ΣPEQ ≥ 1. If we assume that estimates of SS in BIG-C sediments are zero (i.e., none of the observed black particulates were SS), the model relating SS to Cu concentrations is very strong (R^2^ =  0.84). The mean model prediction crosses the Cu PEC of 149 mg Cu/kg dw (i.e., PEQ > 1) at 32% SS, although the prediction interval at this point is very wide (26–851 mg Cu/kg dw; Fig 3). Pearson’s *r* between SS and water depth is 0.42 (95% CI =  0.23–0.59); and Pearson’s *r* between ΣPEQ and water depth is 0.17 (CI =  0.06–0.38).

**Fig 4:**
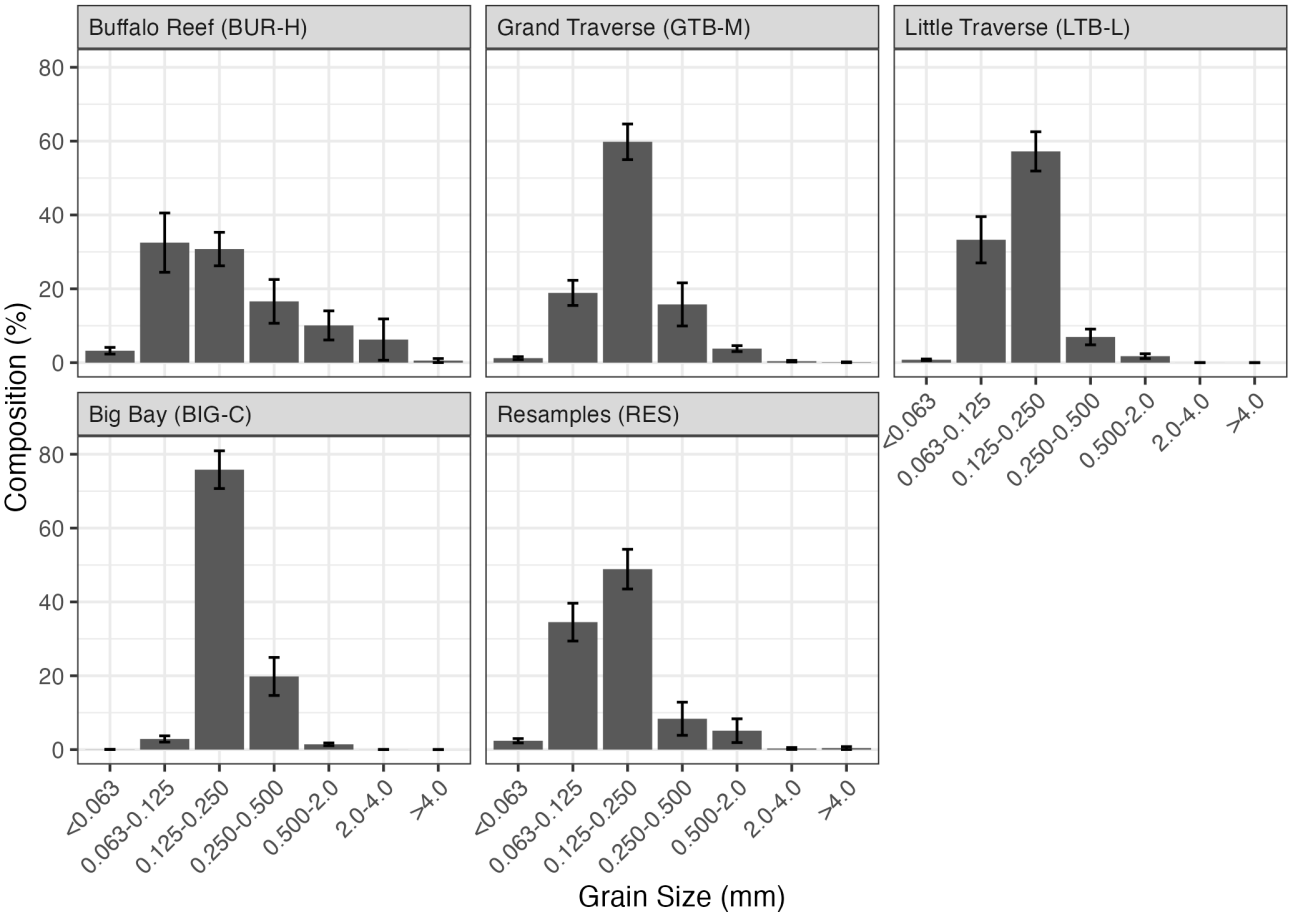
Particle size distribution in sediments collected by petite Ponar at each beach in Lake Superior near the Keweenaw Peninsula, Michigan, 2021. Top of bars are means and vertical lines are standard errors. BUR-H – beach near Buffalo Reef that has been heavily impacted by stamp sands (SS); GTB-M – beach in Grand Traverse Bay south of the Traverse River with some SS contamination; LTB-L – beach in Little Traverse Bay with very little SS; BIG-C- beach in Big Bay (a control site with no SS); RES – samples from sites resampled from Kerfoot et al. [5].

Particle size distributions qualitatively varied across beaches. Compared to LTB-L and BIG-C, BUR-H had a much lower proportion (%) of particles in the 125–250-µm range (medium sand) (Fig 4). BUR-H was the only beach where fine and very fine sands in the 63–125-µm range generally made up the largest proportion of the substrate composition (Fig 4). BUR-H, and RES to a lesser extent, also tended to have a greater proportion of coarse (>500 µm) particles than the other sample groups.

### Variation in benthic communities across beach areas

**Fig 5:**
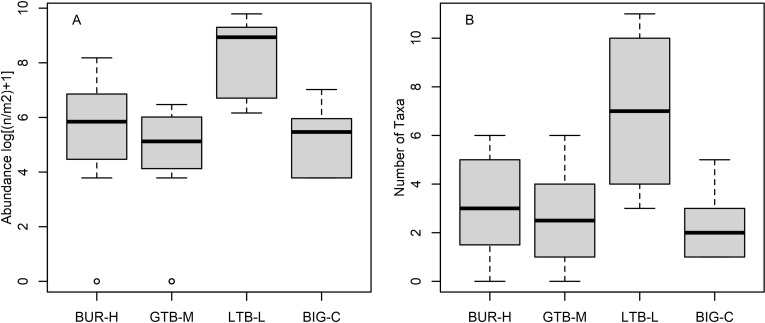
Graphical comparisons of abundance (as number, n, per square meter, m^2^) and number of taxa for benthic invertebrates collected by petite Ponar from beach sites in Lake Superior near the Keweenaw Peninsula, Michigan, 2021. BUR-H – beach near Buffalo Reef that has been heavily impacted by stamp sands (SS); GTB-M – beach in Grand Traverse Bay south of the Traverse River with some SS contamination; LTB-L – beach in Little Traverse Bay with very little SS; BIG-C- beach in Big Bay (a control site with no SS). Boxes encompass the first and third quartiles. The thick black line is the median. The lines (whiskers) show the largest or smallest observation that falls within 1.5 times the box size. Observations that fall outside the lines are shown individually.

**Fig 6:**
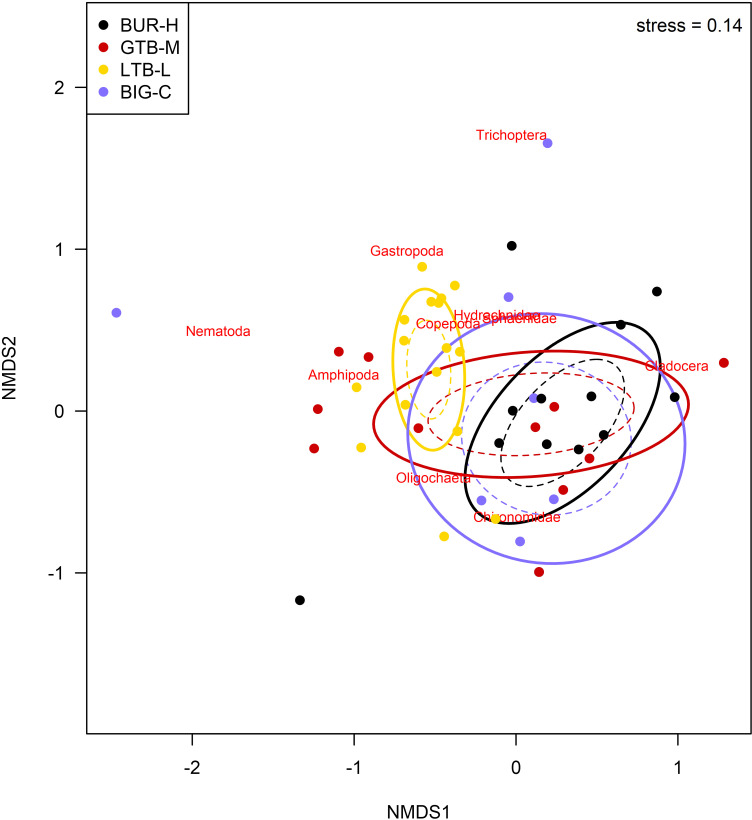
Non-metric, multi-dimensional scaling (NMDS) plot of benthic invertebrate community composition at four beaches) in Lake Superior near the Keweenaw Peninsula, Michigan, 2021. The four beaches varied by stamp sands (SS) impact: BUR-H – beach near Buffalo Reef that has been heavily impacted by SS; GTB-M – beach in Grand Traverse Bay south of the Traverse River with some SS contamination; LTB-L – beach in Little Traverse Bay with very little SS; BIG-C- beach in Big Bay (a control site with no SS). Solid ellipses enclose the centroid of the group within 1 standard deviation, and dashed ellipses enclose the centroid of the group with 95% confidence interval. Individual species are plotted at the point where their distribution is at a theoretical maximum. The two-dimensional stress of the NMDS ordination is 0.14 and the distance matrix was generated using the Bray-Curtis dissimilarity index.

Among the benthic taxa, cladocerans, chironomids, Sphaeriidae, and oligochaetes were present at all beaches (Table 2). Nematodes, copepods, gastropods, and amphipods were absent from BUR-H, but present at one or both control sites (Table 2). Overall, the median abundance of benthic organisms was ~ 3 orders of magnitude higher at LTB-L compared to other beaches (Fig 5, note log scale), driven mainly by large numbers of Harpacticoida copepods. The LTB-L beach also had the greatest number of taxa overall (Table 2, Fig 5). Looking at individual taxa, BUR-H had more *Bythotrephes longimanus* and Holopediidae than the BIG-C site, and fewer Harpacticoida, Chironomidae, Sphaeriidae, Oligochaeta, and Amphipoda than LTB-L (Table 3). GTB-M and BUR-H never had clearly different numbers of these taxa (Table 3). The BUR-H and GTB-M beaches had comparable median diversity index values, whereas BIG-C had the lowest median diversity (Fig 5). The NMDS analyses shows that the benthic community composition at BUR-H has substantial overlap with the communities at both GTB-M and BIG-C, but very little or no overlap with LTB-L (Fig 6); in contrast, LTB-L did overlap with GTB-M and BIG-C.

**Table 2:** Benthic taxa observed at benthic sampling locations in Lake Superior near the Keweenaw Peninsula, Michigan, 2021.

Order/Phylum	Buffalo Reef (BUR-H)	Grand Traverse Bay (GTB-M)	Little Traverse Bay (LTB-L)	Big Bay (BIG-C)	Sites resampled from Kerfoot et al. ([5]; RES)
Cladocera (other than those identified below)	X	X	X	X	X
• *Bythotrephes longimanus*	X	X	X	X	X
• Holopediidae spp.	X	X	X	X	X
Harpacticoida			X	X	
Cyclopoida		X			X
Calanoida					X
Diptera (Chironomidae)	X	X	X	X	X
Trichoptera	X		X	X	X
Gastropoda			X		
Sphaeriidae	X	X	X	X	X
Oligochaeta	X	X	X	X	X
Amphipoda (*Diporeia*)		X	X		X
Hydrachnidia	X		X		
Nematoda		X	X	X	X
Total Orders/Phyla	6	7	10	7	9

**Table 3:** Differences in benthic invertebrate density (log count per m^3^) among beaches, estimated using a generalized linear model at sites in Lake Superior near the Keweenaw Peninsula, Michigan, 2021.

Taxa^1^	^2^ *B*_GTB-M_	*B*_LTB-L_	*B*_BIG-C_	R^2^
*Bythotrephes longimanus*	-8.1 (-52.0, 35.9)	16.1 (-27.8, 60.1)	**-45.7 (-89.6, -1.8)**	0.12
Holopediidae spp.	-301.4 (-615.4, 12.6)	-61.9 (-375.9, 252.1)	**-344.4 (-658.5, -30.4)**	0.10
Harpacticoida*	0 (-1725, 1725)	**4,618 (2,892, 6,343)**	70.0 (-1,655, 1,795)	0.41
Chironomidae	-64.6 (-274.4, 145.2)	**686.2 (476.4, 896.0)**	-10.8 (-220.6, 199.1)	0.53
Sphaeriidae	-29.6 (-122, 63.5)	**185.7 (92.6, 278.8)**	-26.9 (-120.0, 66.2)	0.32
Oligochaeta	-43.1 (-165.6, 262.4)	**570.5 (267.8, 873.1)**	-45.7 (-348.4, 256.9)	0.28
Amphipoda (*Diporeia*)^†^	21.5 (-6.0, 49.0)	**83.4 (55.9, 110.9)**	0 (-27.5, 27.5)	0.44

^1^Trichoptera, Gastropoda, Hydrachnidae and Nematoda occurred at low abundance at all beaches, and therefore could not be modeled using these methods.

^2^Effect sizes (*B*; with 95% confidence intervals) are relative to the Buffalo Reef (BUR-H) beach, which is most affected by stamp sands. Effects sizes in **bold** do not overlap zero, indicating strong support for a difference from BUR-H. GTB-M, Grand Traverse Bay south of Traverse River; LTB-L, Little Traverse Bay; BIG-C, Big Bay; positive effects indicate abundance is greater than at BUR-H.

*Only present at LTB and BIG; no other copepods present in more than 1 sample.

†Only present at GTB and LTB.

### Variation in zooplankton communities across beach areas

**Fig 7:**
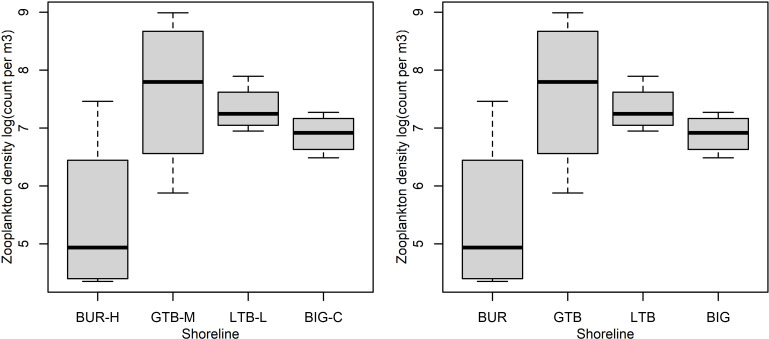
Zooplankton density (count per cubic meter, m^3^) (A) and diversity (B) by beach in Lake Superior near the Keweenaw Peninsula, Michigan, 2021. The beaches varied by impact of stamp sands (SS): BUR-H – beach near Buffalo Reef that has been heavily impacted by stamp sands (SS); GTB-M – beach in Grand Traverse Bay south of the Traverse River with some SS contamination; LTB-L – beach in Little Traverse Bay with very little SS; BIG-C- beach in Big Bay (a control site with no SS). Boxes encompass the first and third quartiles. The thick black line is the median. The lines (whiskers) show the largest or smallest observation that falls within 1.5 times the box size.

**Fig 8:**
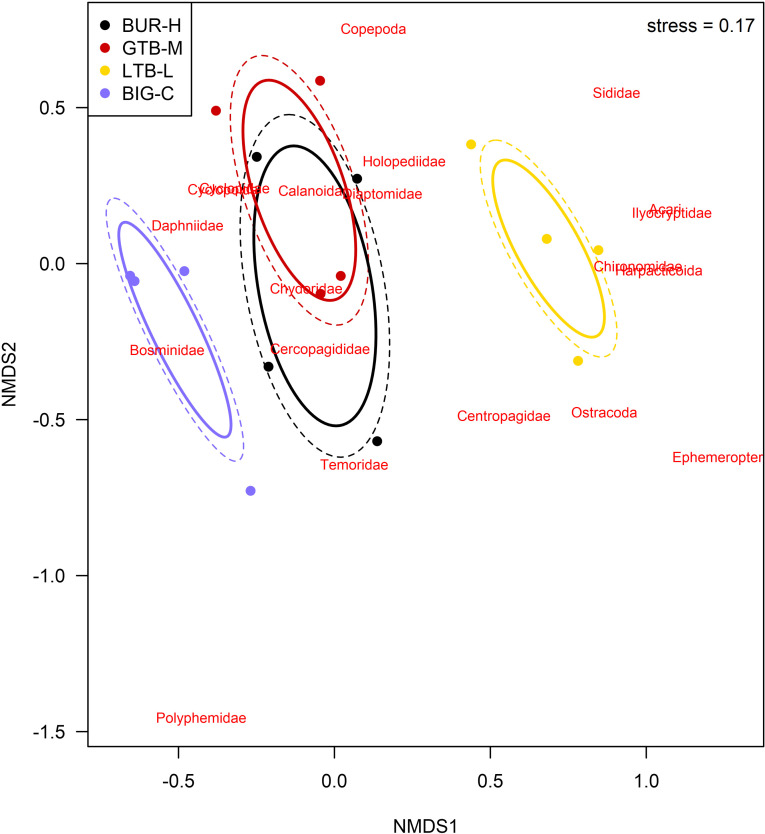
Non-metric, multi-dimensional scaling (NMDS) plot of benthic invertebrate community composition at four beaches) in Lake Superior near the Keweenaw Peninsula, Michigan, 2021. The four beaches varied by stamp sands (SS) impact: BUR-H – beach near Buffalo Reef that has been heavily impacted by SS; GTB-M – beach in Grand Traverse Bay south of the Traverse River with some SS contamination; LTB-L – beach in Little Traverse Bay with very little SS; BIG-C- beach in Big Bay (a control site with no SS). Solid ellipses enclose the centroid of the group within 1 standard deviation, and dashed ellipses enclose the centroid of the group with 95% confidence interval. Individual species are plotted at the point where their distribution is at a theoretical maximum.. The order Cyclopoida and the family Cyclopidae (within the same order) appear in the exact same space on this NMDS plot (which obscured the labeling); therefore, only Cyclopoida is labeled. The two-dimensional stress of the NMDS ordination is 0.17 and the distance matrix was generated using the Bray-Curtis dissimilarity index.

Twenty taxa of invertebrates were identified in the zooplankton samples. Many juvenile copepods were only identified as copepods or to order, but four families of copepods were identified (Centropagidae, Diaptomidae, Temoridae, Cyclopidae; Table 4). Cladocerans included eight families; most other taxa were only identified to sub-class or order (Table 4). A few typically benthic taxa were observed in the plankton nets (e.g., Ephemeroptera once, Chironomidae 10 times; Table 4) and were included in our analyses of the zooplankton. We did not exclude these taxa because they do occur in the pelagic zone at times (e.g., while moving or during disturbance) and because excluding them has no significant effect on the results. The total number of zooplankton taxa observed was greatest at LTB-L (Table 4), where 18 total taxa were identified), whereas other areas had lower diversity (12 taxa at BUR-H, 11 at GTB-M, and 13 at BIG-C). However, individual samples showed considerable variation within sites (Fig 7). Three families of Cladocerans (Ilyocryptidae, Polyphemidae and Sididae), insect taxa (Chironomidae and Ephemeroptera), Acari, and Harpacticoida were absent at BUR-H (Table 4). Zooplankton NMDS plots show considerable overlap between the zooplankton community compositions at BUR-H and GTB-M, but LTB-L and BIG-C were very distinct from the two other beaches (Fig 8).

**Table 4:** Zooplankton taxa observed at the benthic sampling locations in Lake Superior near the Keweenaw Peninsula, Michigan, 2021.

Taxa	Buffalo Reef (BUR-H)	Grand Traverse Bay (GTB-M)	Little Traverse Bay (LTB-L)	Big Bay (BIG-C)
Acari (sub-class)			X	
Cladocera (sub-order)				
■ Bosminidae	X	X	X	X
■ Cercopagididae	X	X		
■ Chydoridae	X	X	X	X
■ Daphniidae	X	X	X	X
■ Holopediidae	X	X	X	
■ Ilyocryptidae			X	
■ Polyphemidae				X
■ Sididae			X	
Chironomidae (Family)			X	X
Ephemeroptera (Order)			X	
Copepoda (Subclass)	X	X	X	
Calanoida (Order)	X	X	X	X
■ Centropagidae	X		X	
■ Diaptomidae	X	X	X	X
■ Temoridae	X	X	X	X
Cyclopoida (Order)	X	X	X	X
Cyclopidae	X	X	X	X
Harpacticoida (Order)			X	X
Ostracoda (Sub-Order)	X		X	
Total Families	13	11	18	11

Total zooplankton (density) was 1–2 orders of magnitude greater at GTB-M, LTB-L and BIG-C relative to BUR-H (Table 5), which is also apparent from the model estimates (Fig 7). Nine taxa (Cladocera and Copepoda) were common to at least three beaches (Table 4), none of which were clearly denser at BUR-H than at other sites (Table 5). As an overall group, Cladocera were more common at all three of the less impacted sites (GTB-M, LTB-L, and BIG-C) than BUR-H, but individual taxa varied (Table 5). For example, Daphniidae were more common at GTB-M than BUR-H, but LTB-L and BIG-C were not clearly distinct from BUR-H. For Copepoda as a group, GTB-M and LTB-L had higher densities than BUR-H, but BIG-C was not clearly distinct (Table 5). Again, when looking at lower taxonomic levels these patterns differed. For example, all of the sites had similar numbers of Temoridae (Table 5).

**Table 5:** Differences in zooplankton density (log count per m^3^) among beaches, estimated using a generalized linear model in Lake Superior near the Keweenaw Peninsula, Michigan, 2021.

Taxa	^1^ *B*_GTB-M_	*B*_LTB-L_	*B*_BIG-C_	R^2^
Total Zooplankton	**2.2 (0.8, 3.6)**	**1.9 (0.5, 3.3)**	**1.5 (0.1, 2.9)**	0.48
Cladocera	**1.1 (0.1, 2.2)**	**2.1 (1.1, 3.1)**	**2.9 (1.9, 3.9)**	0.74
■ Bosminidae	0.2 (-1.2, 1.6)	0.3 (-1.1, 1.8)	**3.3 (1.9, 4.8)**	0.70
■ Chydoridae*	0.3 (-0.9, 1.4)	**3.5 (2.3, 4.6)**	**2.4 (1.2, 3.5)**	0.81
■ Daphniidae	**1.6 (0.05, 3.1)**	0.4 (-1.1, 2.0)	1.1 (-0.4, 2.7)	0.29
■ Holopediidae^†^	**2.6 (1.8, 3.4)**	**3.1 (2.2, 3.9)**	-0.7 (-1.6, 0.1)	0.91
Copepoda	**2.3 (0.8, 3.7)**	**1.5 (0.03, 3.0)**	1.2 (-0.2, 2.7)	0.44
Calanoida	**1.4 (0.3, 2.5)**	**1.5 (0.4, 2.5)**	-0.7 (-1.8, 0.3)	0.66
■ Diaptomidae*	1.4 (-0.1, 2.9)	**2.3 (0.8, 3.8)**	0.5 (-1.0, 2.0)	0.48
■ Temoridae	0.5 (-0.4, 1.4)	0.4 (-0.5, 1.3)	0.3 (-0.6, 1.1)	0.11
Cyclopoida	**3.1 (1.2, 5.0)**	1.8 (-0.1, 3.7)	**2.3 (0.4, 4.2)**	0.47
■ Cyclopidae	**3.0 (1.4, 4.5)**	1.1 (-0.5, 2.7)	1.3 (-0.3, 2.8)	0.53

^1^Effect sizes (*B*; with 95% confidence intervals) are relative to the Buffalo Reef (BUR-H) beach, which is most affected by stamp sands. Effects sizes in **bold** do not overlap zero, indicating strong support for a difference from BUR-H. GTB-M, Grand Traverse Bay south of Traverse River; LTB-L, Little Traverse Bay; BIG-C, Big Bay; positive effect indicate the abundance is greater than BUR-H.

*Not present at BUR-H;

†*Not present at BIG-C.*

### Direct relationships between benthic invertebrates and stamp sand abundance

We used multi-level models to examine direct relationships between invertebrates and SS (i.e., SS, Cu concentrations, and ΣPEQ). For model estimation, the visual estimates of SS at the BIG-C sites were set to zero. We did this for BIG-C sites because surveys, mapping and modeling of SS movement do not suggest that these Gay, MI SS could reach BIG-C at the percentages suggested by visual methods [3] and the metals data indicate it is unlikely these are SS from the Gay, MI pile. Although we originally intended for LTB-L to be a control site as well, the presence of SS here is consistent with the direction of SS movements over the previous decades [3], so we retained these estimates. We also included data from the 16 sites resampled from Kerfoot et al. ( [5]; RES sites). These models treated beach (or RES) as a conditional effect, to account for inherent differences in the suitability of these beaches or sampling areas for benthic invertebrates. The models with the strongest support for total invertebrate density and number of taxa always included water depth (i.e., all models with ΔAIC_C_ < 10), which had a strong positive effect on density and taxa count (Table 6). The strongest model for benthic density included both water depth and Cu concentration; this model had an R^2^ of 0.73, with a marginal R^2^ of 0.28 (Table 6). Similarly, the strongest model for taxonomic richness had an R^2^ of 0.66 and a marginal R^2^ of 0.32 (Table 6). We note that although models with water depth and Cu were ranked highest by ΔAIC_C_, models with ΣPEQ instead of Cu had very similar support (Table 6). Both Cu and ΣPEQ had negative effects on both benthic density and taxonomic richness in these models (Table 6). Because ΣPEQ is a composite variable representing the overall sediment toxicity experienced by the benthic community (including Cu), parsimony indicates ΣPEQ should be conceptually weighted more heavily than Cu alone [40]. Models with only SS had much less support, although the second order polynomial regression (the model with a SS^2^ term) did fit the data better than a null model for both benthic density and taxonomic richness (Table 6).

**Table 6:** Models relating stamp sand (SS) content, copper concentration in the sediment (Cu), the sum of the probable effects quotients (ΣPEQ) for 7 metals in the sediment, and water depth (Depth) to the density and taxonomic richness of the benthic invertebrate community in the Keweenaw Peninsula, Michigan, 2021. BUR-H – beach near Buffalo Reef that has been heavily impacted by SS; GTB-M – beach in Grand Traverse Bay south of the Traverse River with some SS contamination; LTB-L – beach in Little Traverse Bay with very little SS; BIG-C- beach in Big Bay (a control site with no SS); RES – samples from sites resampled from Kerfoot et al. [5].

Response	Model^1^ [with β^2^ (confidence interval)]	ΔAIC_C_	R^2^_Marginal_	R^2^_Conditional_	Standardized intercepts (α)
Benthic density	**Cu × -0.24 (-0.43, -0.06)** + **Depth × 0.57 (0.39, 0.75)**	**0**	**0.28**	**0.73**	**α**_**BUR-H**_ **= 0.10; α**_**GTB-M**_ **= -0.76;** **α**_**LTB-L**_ **= 1.34; α**_**BIG-C**_ **= -0.37;** **α**_**RES**_ **= -0.34**
[log(n m^-2^+1)]	ΣPEQ × -0.25 (-0.43, -0.06) + Depth × 0.59 (0.40, 0.75)	0.1	0.28	0.73	α_BUR-H_ = 0.10; α_GTB-M_ = -0.77; α_LTB-L_ = 1.34; α_BIG-C_ = -0.37; α_RES_ = -0.33
	Depth	4.3	0.27	0.73	
	SS + SS^2^ + Depth	5.7	0.30	0.75	
	SS + Depth	6.0	0.27	0.71	
	SS + SS^2^	26.4	0.22	0.67	
	Cu	30.1	0.10	0.48	
	ΣPEQ	31.0	0.09	0.48	
	*Null*	35.2	–	0.39	
	SS	37.3	<0.01	0.40	
Taxonomic Richness	**Cu × -0.27 (-0.52, -0.04)** + **Depth × 0.47 (0.30, 0.64)**	0	**0.32**	**0.66**	**α**_**BUR**_ **= 1.36; α**_**GTB**_ **= 0.92;** **α**_**LTB**_ **= 1.98; α**_**BIG**_ **= 0.78; α**_**RES**_ **= 1.07**
Species #	ΣPEQ **× ** -0.25 (-0.49, -0.04) + Depth **× ** 0.47 (0.30, 0.65)	0.3	0.31	0.66	α_BUR_ = 1.36; α_GTB_ = 0.92; α_LTB_ = 1.98; α_BIG_ = 0.78; α_RES_ = 1.07
	SS + SS^2^ + Depth	3.1	0.34	0.67	
	Depth	3.5	0.29	0.67	
	SS + Depth	3.5	0.28	0.65	
	SS + SS^2^	23.2	0.24	0.54	
	Cu	28.1	0.13	0.49	
	ΣPEQ	29.3	0.11	0.47	
	*Null*	33.4	–	–	
	SS	35.6	<0.01	0.38	

^1^All models were multi-level models that included a beach grouping variable. Taxonomic richness models used a Poisson distribution because species counts are discrete.

^2^All slope (β) and intercept (α) estimates are standardized. The most strongly supported model is highlighted in bold.

**Fig 9:**
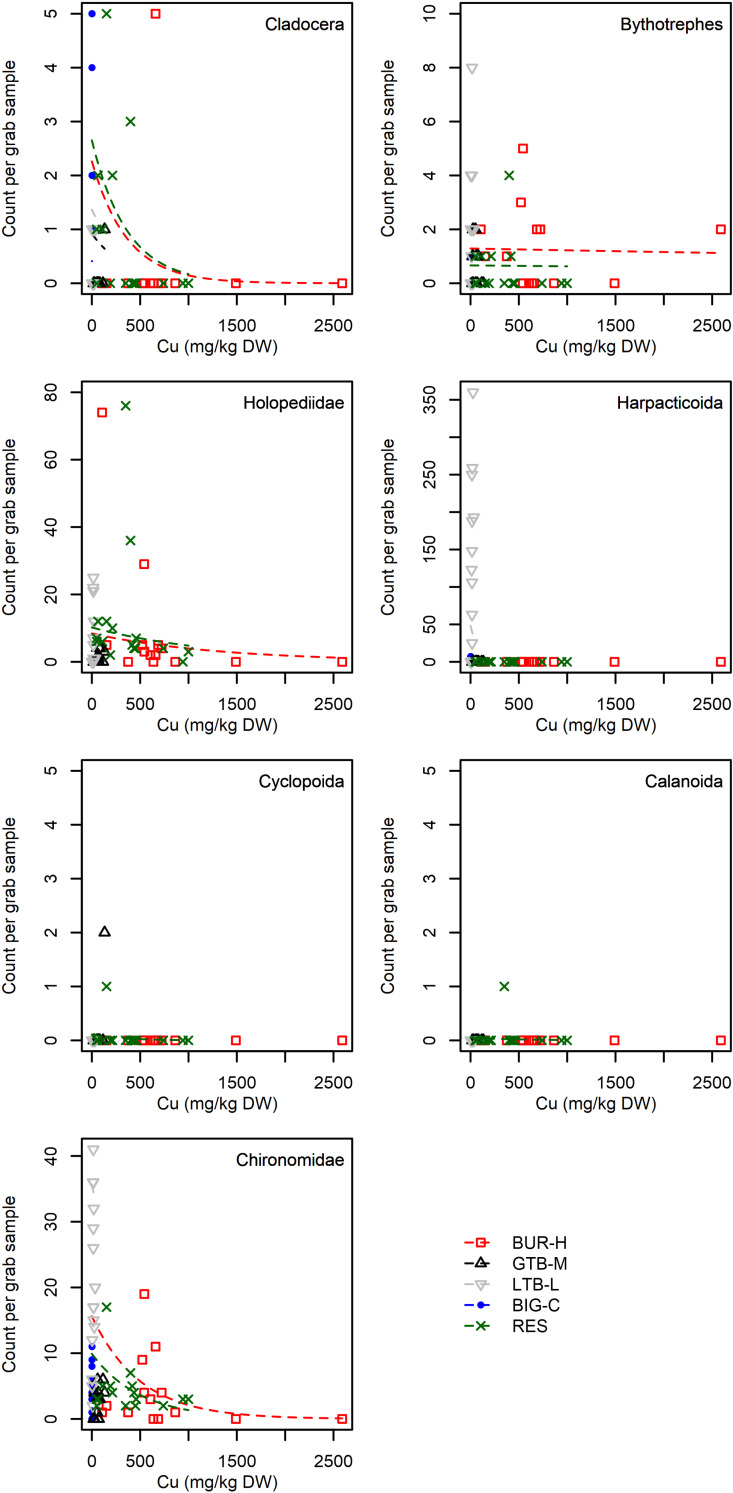
Count per petite Ponar grab sample for seven individual taxa versus copper (Cu) concentration (mg/kg dry weight; DW) in samples collected from Grand Traverse Bay, Little Traverse Bay and Big Bay, Lake Superior, Michigan, 2021. Additional taxa shown in Fig 10. Points are data and dashed lines are the predictions from a multi-level mixed effects models using a negative binomial distribution to predict abundance (Table 8). Predictions are conditional on the taxa and the beach that was sampled, as a result predictions over cover the portion of the gradient in Cu concentration that was present in that beach. As a result, prediction lines for Little Traverse Bay (LTB-L) beach and Big Bay (BIG-C) beach are very short, as these beaches have very low Cu concentrations compared to the other beach categories. BUR-H – beach near Buffalo Reef that has been heavily impacted by stamp sands (SS); GTB-M – beach in Grand Traverse Bay south of the Traverse River with some SS contamination; LTB-L – beach in Little Traverse Bay with very little SS; BIG-C- beach in Big Bay (a control site with no SS); RES – samples from sites resampled from Kerfoot et al. [5].

**Fig 10:**
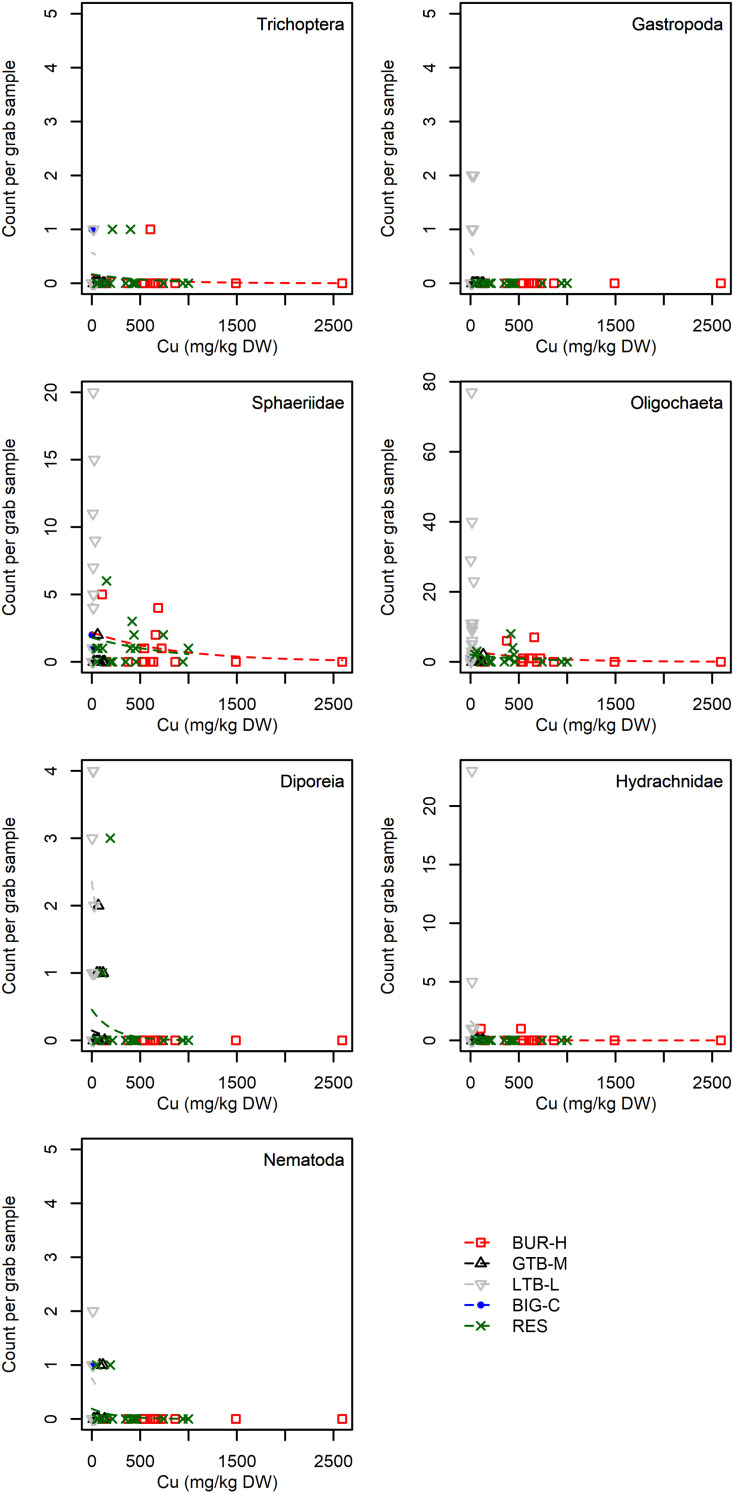
Count per petite Ponar grab sample for seven individual taxa versus copper (Cu) concentration (mg/kg dry weight; DW) in samples collected from Grand Traverse Bay, Little Traverse Bay and Big Bay, Lake Superior, Michigan, 2021. Additional taxa shown in Fig 9. Points are data and dashed lines are the predictions from a multi-level mixed effects models using a negative binomial distribution to predict abundance (Table 8). Predictions are conditional on the taxa and the beach that was sampled, as a result predictions over cover the portion of the gradient in Cu concentration that was present in that beach. As a result, prediction lines for Little Traverse Bay (LTB-L) beach and Big Bay (BIG-C) beach are very short, as these beaches have very low Cu concentrations compared to the other beach categories. BUR-H – beach near Buffalo Reef that has been heavily impacted by stamp sands (SS); GTB-M – beach in Grand Traverse Bay south of the Traverse River with some SS contamination; LTB-L – beach in Little Traverse Bay with very little SS; BIG-C- beach in Big Bay (a control site with no SS); RES – samples from sites resampled from Kerfoot et al. [5].

When we included individual taxa as a random effect, the overall model structure was similar to the benthic density model, but the marginal R^2^ value was lower (0.16), and the effect of Cu was stronger than the effect of depth (Table 7). The effect size of Cu varied substantially across taxa, although for some taxa there were very few observations (Table 8, Fig 9, Fig 10). Standardized model effect sizes indicate that some taxa were more strongly affected by Cu than depth (Table 8): Harpacticoida, Gastropods, Diporeia, Hydrachnidia and Nematoda all had much larger negative effects from Cu than positive effects from depth (Table 8). Of these, Harpacticoida (a benthic copepod) and *Diporeia* (a semi-benthic amphipod) are known to be important for larval fish. By contrast, model estimates indicate the invasive taxa *Bythotrephes longimanus* was not affected by Cu concentration, but was moderately affected by depth (Table 8, Fig 9). Other Cladocerans were also strongly influenced by depth, although they were also affected by Cu concentration to a lesser degree (Table 8, Fig 9). Cyclopoida and Calanoida copepods were modeled as having stronger depth effects, but these estimates are based on just 1 or 2 observations (Table 8; Fig 9). Simlarly, the model indicates Gastropods were strongly affected by Cu concentrations, although there are only a few observations (Table 8; Fig 9). Chironomids and Oligochaetes had very similar effect sizes from both Cu concentration and depth (Table 8, Fig 9, Fig 10). By far the strongest model effect was of Cu concentrations on Harpacticoida copepods, which were never observed at a Cu concentration above 33.9 mg/kg DW and only occurred in the low impact (LTB-L) and control (BIG-C) beaches.

**Table 7:** Comparison of models relating stamp sand (SS) content, copper concentration in the sediment (Cu), the sum of the probable effects quotients (ΣPEQ) for 7 metals in the sediment, and water depth (Depth) to the presence of benthic invertebrate community sampled from beaches in Lake Superior, Michigan, 2021. Models are ranked using Akaike’s information criterion, corrected for small sample size (ΔAIC_C_). Models were generalized multi-level models that used a negative binomial distribution to estimate the number of individuals in a petite Ponar grab. Standardized coefficients and intercept are reported for the best model. Unstandardized coefficients by taxa are reported in Table 8.

Response	Model^1^ [with β^2^ (confidence interval)]	ΔAIC_C_	R^2^_Marginal_	R^2^_Conditional_	Standardized intercepts
Community composition	Cu ^*^ **-1.31 (-2.43, -0.51)** + Depth ^*^ **1.13 (0.75, 1.50)**	**0**	**0.16**	**0.63**	**α**_**BUR**_ **= 0.86; α**_**GTB**_ **= -1.16; α**_**LTB**_ **= 2.40; α**_**BIG**_ **= -1.08; α**_**RES**_ **= -0.60**
Count per petite Ponar sample per taxa	ΣPEQ + Depth	2.5	0.14	0.61	
	SS + SS^2^ + Depth	12.2	0.10	0.56	
	SS + Depth	15.2	0.08	0.53	
	Depth	35.3	0.08	0.50	
	SS + SS^2^	82.8	0.03	0.40	
	Cu	92.2	0.09	0.44	
	ΣPEQ	96.3	0.07	0.41	
	SS	110.4	0.001	0.30	
	Null	126.1	–	–	

The most strongly supported model is highlighted in bold.

**Table 8:** Results of multi-level model (MLM) showing unscaled effect sizes of stamp sands (SS, as a percentage; %) and depth (in meters) on the benthic invertebrate community composition at several beaches in Lake Superior, Michigan. The inferred SS percentage at the maximum likelihood of presence for each taxa was calculated by solving for the SS percentage at the vertex of the parabola defined by the SS and SS^2^ effect sizes. At several beaches, the maximum %SS never occurred. BUR-H – beach near Buffalo Reef that has been heavily impacted by SS; GTB-M – beach in Grand Traverse Bay south of the Traverse River with some SS contamination; LTB-L – beach in Little Traverse Bay with very little SS; BIG-C- beach in Big Bay (a control site with no SS); RES – samples from sites resampled from Kerfoot et al. [5]. The strongest standardized effect for each taxa is highlighted in bold.

Order/Phylum	Intercept	β_Cu_ (standardized)	Cu (mg/kg DW)	β_Depth_ (standardized)	Depth (m)
*Fixed Effects (plus random intercept by beach)*	BUR-H = 0.82; GTB-M = -1.20; LTB-L = 2.34; BIG-C = -1.12; RES = -0.62	-1.31	*-0.0035*	1.13	*0.41*
*Random Effects (by taxa)*					
Cladocera (other than those identified below)	-3.81	-1.10	-0.0027	**1.88**	0.67
• *Bythotrephes longimanus*	-1.57	-0.03	-0.0001	**0.56**	0.23
• Holopediidae spp.	-2.36	-0.32	-0.0008	**1.89**	0.69
Harpacticoida	0.63	**-4.46**	-0.0112	0.62	0.27
Cyclopoida	-5.70	-1.02	-0.0031	**1.64**	0.53
Calanoida	-6.20	-0.47	-0.0019	**1.41**	0.43
Diptera (Chironomidae)	0.39	-0.81	-0.0020	**0.84**	0.33
Trichoptera	-5.17	-0.62	-0.0017	**1.55**	0.52
Gastropoda	-4.11	**-1.73**	-0.0050	0.94	0.34
Sphaeriidae	-2.23	-0.47	-0.0011	**1.12**	0.42
Oligochaeta	-0.71	**-0.63**	-0.0016	0.62	0.24
Amphipoda (*Diporeia*)	-2.37	**-1.81**	-0.0046	0.84	0.33
Hydrachnidia	-3.07	**-1.64**	-0.0043	0.88	0.33
Nematoda	-3.69	**-1.57**	-0.0042	0.98	0.36

## Discussion and conclusions

We observed, as did Kerfoot et al. [3,5], that SS and/or metals associated with SS are associated with changes in benthic and pelagic invertebrate communities. These mining by-products are rich in Cu, and models identified Cu as having a negative association with benthic density, taxa number and several individual taxa. *Diporeia* and benthic copepods are important prey items for larval fish, and these taxa were among the most sensitive to Cu concentrations in the sediment. These taxa were also less abundant at the SS covered BUR-H beach compared to beaches with little or no SS. Moreover, zooplankton density was orders of magnitude lower at BUR-H than at the other beaches. There are multiple mechanisms by which SS can impair benthic invertebrates (e.g., toxicity from metals, abrasion, physical blockage of habitat). The concentration of Cu (or ΣPEQ, the combination of Cu and other metals sourced from the SS) was more strongly associated with declines in overall benthic abundance, diversity and community composition than were visual estimates of SS percentage, indicating that exposure to Cu (and the resulting uptake and toxicity) may be the primary cause of the effects on benthic macroinvertebrates. Direct Cu toxicity is probably also the major driver of effects on zooplankton (discussed further below).

Here we used a log-normal model structure (by using a log transformation) to model associations between measurements of SS and invertebrate abundance and diversity. Kerfoot et al. [3] noted that this non-linearity indicates that the toxicity of SS varies across the range of SS percentages. Kerfoot et al. [3] also observed that SS that have traveled farther appear to have lower Cu concentration. As lower concentrations of SS correspond to sites farther from the original SS source, this would explain why relationships between SS and Cu or SS and invertebrate community directly are non-linear. However, our model was non-linear even when using Cu data directly. This implies that the effect of Cu and other metals themselves are non-linear and synergistic effects are likely occurring in the field.

Unlike benthic taxa, zooplankton are less likely to be affected by physical abrasion or filling of interstitial spaces in the sediment. This means SS effects are likely driven by metal leaching from sediments either directly (via toxicity) or indirectly (via food web effects). Relative to other sites, BUR-H had fewer zooplankton cladocerans and copepods (up to 2 orders of magnitude fewer). This varied among the individual taxa within those larger groups, but as a food resource for larval fish, the zooplankton community as a whole was clearly affected at BUR-H. Cladocerans and copepods were the most abundant zooplankton taxa, and copepods were the most abundant benthic invertebrate. We know that not only are copepods and other aquatic invertebrates and vertebrates sensitive to dissolved Cu in the water column (e.g., [46,47]), but that waterborne Cu can result in major changes in the planktonic community structure [48]. We do not have metals data for water at our study sites to demonstrate potential leaching of Cu into the water column. However, we believe it is reasonable to conclude that this is the source of reduced abundance at BUR-H. Numerous invertebrate taxa are sensitive to Cu exposure (e.g., [49]), either directly or via trophic transfer mechanisms (e.g., [50]). Water-only metals exposures have previously been shown to poorly predict biological effects from sediment toxicity, including that of sediments collected in the Keweenaw Waterway and elsewhere [51,52], but the data here indicate these biological effects can be severe for the zooplankton community’s overall abundance.

Our data, although limited to a single year, indicates that literature-based definitions of sediment toxicity (i.e., exceedances of PECs, ΣPEQ ≥ 1) may not be protective of community structure and diversity in these beach areas. Most BUR-H sites had Cu concentrations above the PEC, while all of the GTB-M and LTB-L sites had concentrations below the Cu PEC. However, the overall picture from the invertebrate data here indicates BUR-H and GTB-M are more similar to each other than to LTB-L. We hypothesize that GTB-M is already experiencing effects from SS-derived Cu, even though this site mostly has concentrations below the PEC threshold, and therefore Cu concentrations would need to be kept lower than the PEC to prevent negative effects. In addition to the PEC, MacDonald et al. [30] also reported consensus-based threshold effect concentrations (TECs), below which harmful effects are unlikely to be observed. The TEC value for Cu (31.6 mg/kg dw) was exceeded in 100% of the BUR-H and RES samples, as well as in 11 of the GTB-M samples (69%) and 1 LTB-L (6%) sample. The Cu TEC threshold may be a better indicator of effects in our study data than the PEC, with GTB-M and BUR-H on one side of the TEC and LTB-L on the other, consistent with the invertebrate community data. Although our data are consistent with GTB-M having already suffered impacts from Cu derived from SS, we lack a before-after dataset that would make a compelling case for this. The possibility exists that LTB-L has always been very different than GTB-M and BUR-H, even prior to SS arrival.

Predicting and modeling Cu from SS concentrations is challenging. Concentrations of Cu in the Gay, Michigan, SS have been measured in several studies, but the exact leaching and weathering processes that occur and potentially contribute to biological effects once the sands enter and drift within Lake Superior are largely unknown. Soil sampling (i.e., onshore sampling of SS deposit areas) by the Michigan Department of Environmental Quality [16] indicated a mean Cu concentration of 2,683 mg/kg (range 1,500–13,000) in soils collected from areas closest to the conveyor that was used to transport sands into Lake Superior at the time of mill operation. If we were to extrapolate using the mean Cu concentration from those undisturbed soils, then this would indicate that Cu PECs would be exceeded when SS are more than about 5% (range 1–10%). This is less than the lowest observed value where Cu PEC was exceeded in our dataset (15.3%), indicting the SS we observed had lower Cu content than the original pile. Another deposit area, which represented an onshore area in which SS that had eroded from the main pile were deposited, had a mean Cu concentration of 1,443 mg/kg (range 710–5,300 [53];). These SS had experienced more movement and weathering, and either these processes had leached Cu from the SS or particles with less Cu are more mobile [3]. These weathered SS would need to make up 10% (3–21%) of the sands at a site to reach the Cu PEC, which is closer to the low end of our observations. To our knowledge, this is the first time that SS have been observed to make up > 10% of the sand at any LTB-L sites; indeed, we observed SS > 10% a total of 4 times. Some of these LTB-L sands could be manganese sands previously discussed in Kerfoot et al [3], but in Kerfoot et al., the dark manganese sands made up, on average, just 1.8% of grain counts in Grand Traverse Bay. However, darker sands could be more prevalent in other areas (as we observed in Big Bay), so visual estimation of SS may not be ideal everywhere. Still, given the relatively low concentrations of SS required to reach the Cu PEC and given the Cu PEC may not be protective of the invertebrate communities, this may indicate that the LTB-L site may be in imminent danger of experiencing impacts to the invertebrate community.

Our results indicate that continued direct measurements of Cu and other metals in SS collected from Lake Superior would be important because extrapolation of Cu from SS collected from previous onshore soil studies could misrepresent *in situ* conditions for effects determinations and remedial purposes [3]. Additionally, SS heterogeneity within each beach likely results in localized exposure conditions to fish eggs, larvae, and the benthos. In other words, the benthos, including larval fish, could be exposed to locally elevated metals concentrations due to differing SS within their home ranges, based on relative immobility compared to adult fish. Localized effects are supported by our models, which indicated that Cu concentrations in the sediments and water depth were more critical for benthic density and taxonomic richness than SS.

The data here are limited in several ways that could influence interpretation. Primarily, these data come from a single year of sampling, and many factors could vary year-to-year that could affect these results. Our study design included two control beaches (LTB-L and BIG-C), but our actual data suggested that LTB-L might already be contaminated by SS and BIG-C has a very distinct orientation and fetch compared to the other sites. As a result, neither of these beaches are perfect controls. Further, any field collected observational data is potentially subject to cryptic environmental variation that we did not identify.

Still, the data here indicates BUR-H has fewer zooplankton and reduced densities of some important benthic taxa relative to beaches farther afield from Gay, Michigan. In the context of prior literature, these data support the assessment that SS inundation has resulted in biological and community effects. Our models indicate that > 15% SS is associated with sediment toxicity due to Cu; many of our GTB-M sites have reached this threshold. Additional studies of SS toxicity, both in terms of SS thresholds (particularly given the possibility that SS have begun reaching LTB-L) and associated biological effects to the benthos and larval fish are warranted. Land managers could consider differences in sensitivities among different life stages and taxa (e.g., [52],) and use multi-pronged biological and chemical investigations, including quantification of sediment toxicity modifiers (e.g., organic carbon, particle size [50,54];) to (1) establish site-specific sediment quality assessment frameworks for the Keweenaw Peninsula, and (2) evaluate remedial options to restore the critical nursery habitat at Buffalo Reef. Larval fish raised on Buffalo Reef and growing on nearby beaches are likely to be affected by the declines in early life-stage food resources (e.g., zooplankton) that we observed here.

## Supporting information

S1 Table Comparison of metal concentrations among four beaches potentially impacted by a stamp sands (SS) source near Gay, MI. The most highly impacted beach is near Buffalo Reef (BUR-H). Another beach in Grand Traverse Bay south of the Traverse River is moderately impacted by SS (GTB-M) and very low SS are believed to occur at a beach in nearby Little Traverse Bay (LTB-L). We also sampled a control beach, Big Bay (BIG-C). We used the Peto-Peto test, which is a non-parametric test appropriate when some data are below the quantification limit and the data are non-normally distributed. Medians (standard deviation) are in units of mg/kg dw and were estimated using Kaplan-Meier methods to account for occasions when values were below the limit of quantification [29]. The Peto-Peto test indicated concentration differences between beaches having different letters.
(DOCX)
