# Supplementary material for: Effect of copper mill waste material on benthic invertebrates and zooplankton diversity and abundance
Source: PLoS One. 2025 Mar 3;20(3):e0318980. doi: 10.1371/journal.pone.0318980 (PMC11875367; doi:10.1371/journal.pone.0318980)
Supplement: S1 Table — The most highly impacted beach is near Buffalo Reef (BUR-H). Another beach in Grand Traverse Bay south of the Traverse River is moderately impacted by SS (GTB-M) and very low SS are believed to occur at a beach in nearby Little Traverse Bay (LTB-L). We also sampled a control beach, Big Bay (BIG-C). We used the Peto-Peto test, which is a non-parametric test appropriate when some data are below the quantification limit and the data are non-normally distributed. Medians (standard deviation) are in units of mg/kg dw and were estimated using Kaplan-Meier methods to account for occasions when values were below the limit of quantification [29]. The Peto-Peto test indicated concentration differences between beaches having different letters. (DOCX) [file pone.0318980.s001.docx]

Table S1. Comparison of metal concentrations among four beaches potentially impacted by a stamp sands (SS) source near Gay, MI. The most highly impacted beach is near Buffalo Reef (BUR-H). Another beach in Grand Traverse Bay south of the Traverse River is moderately impacted by SS (GTB-M) and very low SS are believed to occur at a beach in nearby Little Traverse Bay (LTB-L). We also sampled a control beach, Big Bay (BIG-C). We used the Peto-Peto test, which is a non-parametric test appropriate when some data are below the quantification limit and the data are non-normally distributed. Medians (standard deviation) are in units of mg/kg dw and were estimated using Kaplan-Meier methods to account for occasions when values were below the limit of quantification [30]. The Peto-Peto test indicated concentration differences between beaches having different letters.

|  | BUR-H | GTB-M | LTB-L | BIG-C |
| --- | --- | --- | --- | --- |
| Arsenic | 1.74 (0.58) a,b | 1.16 (0.21) c | 1.48 (0.38) b | 2.07 (0.62) a |
| Cadmium | 0.09 (0.03) a | 0.03 (0.01) b | 0.03 (0.01) b | Below LOQ† |
| Cobalt | 13.9 (7.0) a | 1.98 (1.22) b | 0.85 (0.42) c | 0.88 (0.32) b, c |
| Chromium | 16.4 (10.1) a | 3.73 (1.99) b | 2.29 (1.02) b | 3.16 (2.69) b |
| Copper | 745 (601) a | 60.1 (32.8) b | 14.0 (8.4) c | 1.70 (0.30) d |
| Iron (x 10^3^) | 10 (4) a | 3.3 (1.4) b | 2.5 (1.1) c | 5.7 (3.3) d |
| Lead | 2.60 (1.20) a | 1.40 (0.70) a | 1.60 (0.60) a | 1.80 (1.10) a |
| Manganese | 243 (116) a | 48.2 (25.4) b | 41.6 (25.4) b | 45.6 (19.5) b |
| Nickel | 16.5 (7.8) a | 3.30 (1.80) b | 2.00 (1.80) b | 1.80 (0.58) b |
| Selenium | 0.22 (0.07) a | 0.11 (0.03) b | 0.11 (0.02) b | 0.14 (0.05) b |
| Zinc | 47.1 (22.8) a | 9.00 (4.70) b | 6.00 (2.30) c | 7.90 (1.70) b |

† A single BIG site had a positive detection.
